# Behavioural relevance of redundant and synergistic stimulus information between functionally connected neurons in mouse auditory cortex

**DOI:** 10.1186/s40708-023-00212-9

**Published:** 2023-12-05

**Authors:** Loren Koçillari, Marco Celotto, Nikolas A. Francis, Shoutik Mukherjee, Behtash Babadi, Patrick O. Kanold, Stefano Panzeri

**Affiliations:** 1 Istituto Italiano Di Tecnologia, 38068 Rovereto, Italy; 2 Department of Excellence for Neural Information Processing, Center for Molecular Neurobiology (ZMNH), University Medical Center Hamburg-Eppendorf (UKE), Falkenried 94, 20251 Hamburg, Germany; 3 Department of Neurophysiology and Pathophysiology, University Medical Center Hamburg-Eppendorf (UKE), 20246 Hamburg, Germany; 4 Department of Pharmacy and Biotechnology, University of Bologna, 40126 Bologna, Italy; 5 Department of Biology and Brain and Behavior Institute, University of Maryland, College Park, MD 20742 USA; 6 Department of Electrical and Computer Engineering and Institute for Systems Research, University of Maryland, College Park, MD 20742 USA; 7 Department of Biomedical Engineering and Kavli Neuroscience Discovery Institute, Johns Hopkins University, Baltimore, MD 21205 USA

**Keywords:** Functional connectivity, Redundancy, Synergy, Auditory perception

## Abstract

Measures of functional connectivity have played a central role in advancing our understanding of how information is transmitted and processed within the brain. Traditionally, these studies have focused on identifying redundant functional connectivity, which involves determining when activity is similar across different sites or neurons. However, recent research has highlighted the importance of also identifying synergistic connectivity—that is, connectivity that gives rise to information not contained in either site or neuron alone. Here, we measured redundant and synergistic functional connectivity between neurons in the mouse primary auditory cortex during a sound discrimination task. Specifically, we measured directed functional connectivity between neurons simultaneously recorded with calcium imaging. We used Granger Causality as a functional connectivity measure. We then used Partial Information Decomposition to quantify the amount of redundant and synergistic information about the presented sound that is carried by functionally connected or functionally unconnected pairs of neurons. We found that functionally connected pairs present proportionally more redundant information and proportionally less synergistic information about sound than unconnected pairs, suggesting that their functional connectivity is primarily redundant. Further, synergy and redundancy coexisted both when mice made correct or incorrect perceptual discriminations. However, redundancy was much higher (both in absolute terms and in proportion to the total information available in neuron pairs) in correct behavioural choices compared to incorrect ones, whereas synergy was higher in absolute terms but lower in relative terms in correct than in incorrect behavioural choices. Moreover, the proportion of redundancy reliably predicted perceptual discriminations, with the proportion of synergy adding no extra predictive power. These results suggest a crucial contribution of redundancy to correct perceptual discriminations, possibly due to the advantage it offers for information propagation, and also suggest a role of synergy in enhancing information level during correct discriminations.

## Introduction

Functional connectivity (FC) has emerged as a mainstream concept and a fundamental tool for understanding how brain networks process and communicate information, and how functional interactions between networks or between neurons shape the dynamics and function of the brain [1–10]. Traditional measures of FC have mainly focused on redundant connectivity, by measuring (for example, through linear cross-correlations) the similarity of activity between different sites. However, recent studies have begun to highlight the importance of another notion of FC: synergistic connectivity [11–15]. This notion of connectivity focuses on how variations of the interaction between activity at different sites or between activity of different neurons create information that is not present at each site or in each neuron alone [4, 14–16]. Whilst the presence and merits of redundant connectivity have been extensively documented [1, 3, 17, 18], it remains unclear whether synergistic interactions are prominent and how they contribute to cognitive function.

Correlated activity is present in multiple spatial scales, from brain areas to local networks. Consequently, an additional question pertains to the spatial scale at which both redundant and synergistic interactions are expressed. Most previous studies of FC investigated it at a coarse scale, such as that obtained with non-invasive measures of neural activity, such as fMRI or EEG, that do not have single-neuron resolution [19–23]. However, the organization of FC at the finer spatial scale of population recordings with single-neuron resolution is less understood, and its relationship to redundancy or synergy of information encoding at this finer scale has been considered only seldom [24].

In this study, we address some of these open questions regarding synergistic and redundant FC. First, we address their relationship with respect to a widely used directed FC measure, Granger Causality (GC) [25, 26], between the activities of different neurons. This measure of FC is interesting because, unlike simple measures of FC based on cross-correlation, it considers not only the similarity of activity but also the strength and directionality of information transmission. GC, as well as other measures implementing the Wiener–Granger Causality principle [27], can, in principle, capture redundant FC because the process of transmission entails sharing of information between the sending and the receiving site [28, 29]. However, it can also correspond to synergistic FC. For example, if transmission varies across sensory stimuli, FC can create sensory information not available in each site individually. Second, we use precise information-theoretic measures to quantify redundancy and synergy related to the encoding of behaviourally relevant sensory variables (in this case, features of auditory stimuli). These measures, based on the theory of Partial Information Decomposition (PID) [30, 31], have the advantage of separating redundancy from synergy, something that simpler measures [32] used in recent studies [33, 34] cannot achieve. Third, we study synergistic and redundant stimulus information with single-neuron resolution, using the primary auditory cortex (A1) of the mouse brain as an experimental model. Fourth, we explore the potential impact of synergy and redundancy on sensory processing by studying how they vary between cases of correct and incorrect perceptual discrimination.

Part of this work has been presented at the 16th International Conference of Brain Informatics and published as a conference paper [35].

## Experimental task and single-neuron stimulus information

**Fig. 1:**
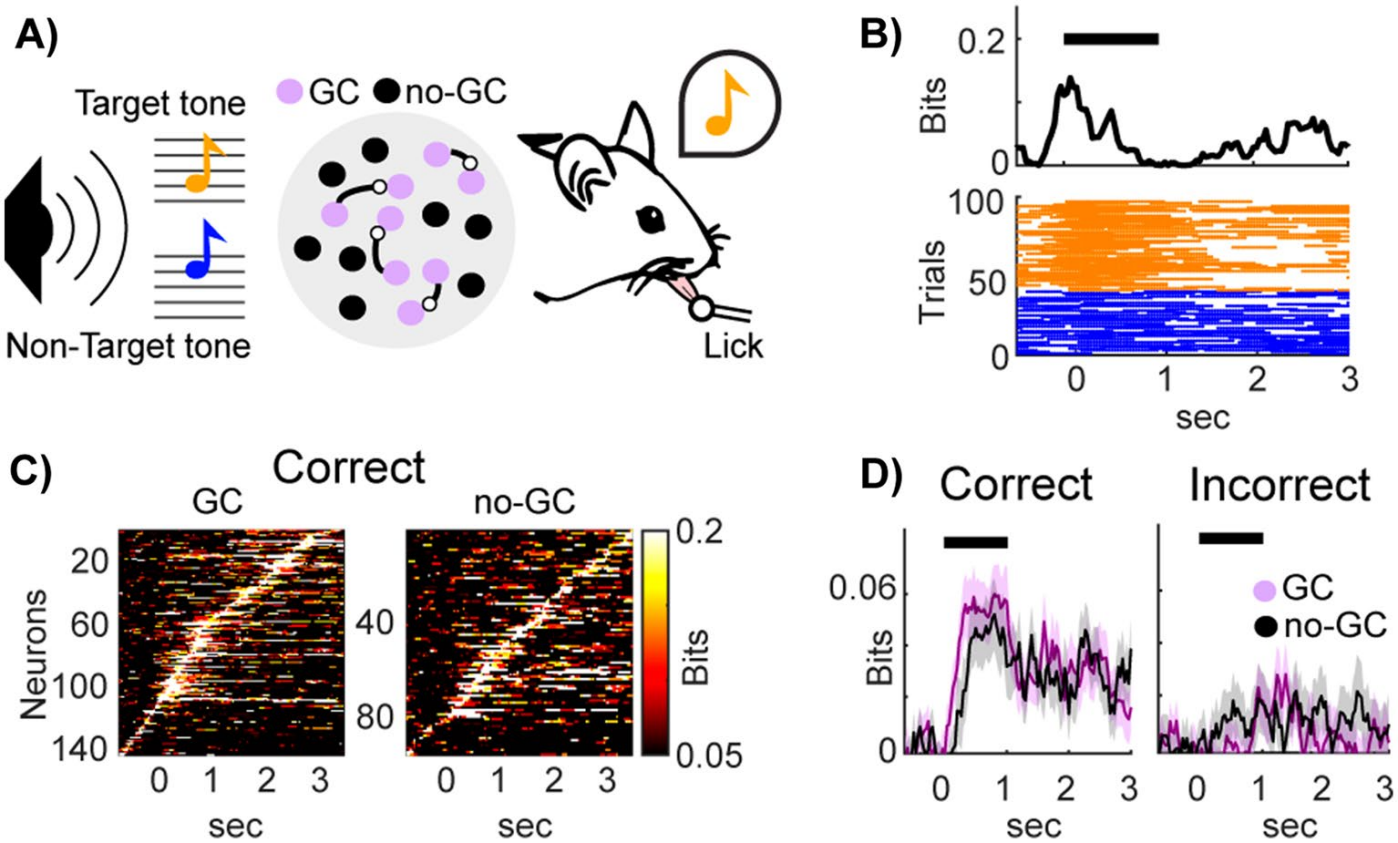
Stimulus information in mouse auditory cortex during a tone discrimination task.** A** Mice performed a go/no-go tone discrimination task whilst the activity of A1 L2/3 neurons were recorded with two-photon calcium imaging. In response to a target tone (low-frequency, in orange) mice had to lick a waterspout and not to lick for non-target tones (high-frequency, in blue). Granger causality analysis revealed sparsely connected networks of cells in A1 L2/3 [34]. We classified neurons as GC (purple) and no-GC (black) depending on whether they formed a GC link. **B** Top: Example of the stimulus information time-course for a single neuron. We computed the time-resolved stimulus information as the mutual information between the auditory stimuli (low-/high-frequency tones) and the spiking activity across trials. Bottom: The traces of the deconvolved spiking activity in each trial, colour-coded based on the tone presented in each trial. The time axes are referenced based on the stimulus onset. **C** Stimulus information time-course for GC neurons (left map) and no-GC neurons (right map) in correct trials only. We then sorted the peaks of stimulus information for each neuron to tile the trial time. **D** Stimulus information for GC (in purple) and no-GC (black) neurons computed separately in trials with correct and incorrect choices. Full lines denote the mean across neurons, whilst the shaded areas denote the SEM across neurons

To investigate the relationship between FC and the presence of synergistic and redundant information with single-neuron resolution, we focused on the activity of the mouse primary auditory cortex during a sound discrimination task. We reanalysed a previously published dataset [34] in which the activity of several tens to a few hundreds of neurons was recorded simultaneously using in vivo two photon calcium imaging from A1 L2/3 neurons in transgenic mice during a pure-tone discrimination task (Fig. 1A).

The experimental task was structured as follows. After a pre-stimulus interval of 1 s, head-fixed mice were exposed to either a low-frequency (7 or 9.9 kHz) or a high-frequency (14 or 19.8 kHz) tone for a period of 1 s. Mice were trained to report their perception of the sound stimulus by their behavioural choice, which consisted of licking a waterspout in the post-stimulus interval (0.5–3 s from stimulus onset) after hearing a low-frequency tone (target tones) and holding still after hearing high-frequency tones (non-target tones). Calcium imaging was used to continuously acquire the fluorescence signals from individual A1 L2/3 neurons during the task with a sampling frequency of 30 Hz.

We used Shannon mutual information [36, 37] to compute the stimulus information carried by each neuron about the stimulus category (low- vs high-frequency tones) in each imaging time frame (Fig. 1B, top plot). Stimulus information is defined as follows:

1 $$SI\left(S;{R}_{i}\right)={\sum }_{s \in S, {r}_{i}\in R}\,p\left(s,{r}_{i}\right)lo{g}_{2}\frac{p\left(s,{r}_{i}\right)}{p\left(s\right)p\left({r}_{i}\right)},$$

where *i* indexes the neurons and $p(s,{r}_{i})$ denotes the joint probability of observing in a given trial the activity ${r}_{i}$ of neuron *i* and the value *s* of the stimulus variable *S*. $p({r}_{i})={\sum }_{s}p(s,{r}_{i})$ and $p\left(s\right)=\sum_{{r}_{i}}p(s,{r}_{i})$ are the marginal probabilities.

The activity ${r}_{i}$ of neuron *i* was inferred following the same approach described in [34]. In brief, we first deconvolved the single-trial calcium fluorescence traces of each neuron to infer the spiking activity (Fig. 1B, bottom). We then aligned neural activity of each trial to the stimulus onset. We used a sliding window approach (windows of 10 time frames with time-steps of 1 time frame) to binarize the deconvolved spiking activity of each window into 0 and 1, where 1 denotes spiking activity higher than 0. We then computed the time-resolved stimulus information on these binarized neural responses using the probabilities $p(s,{r}_{i})$ obtained empirically from the data and plugging them into the information theoretic equations [38]. Finally, we subtracted the average stimulus information computed in the pre-stimulus interval from the stimulus information time-courses, which enabled us to correct for the systematic error (or bias) in the information estimate due to the limited number of trials [39].

Following our previous study [34], we first analysed the entire dataset (2792 neurons recorded from 34 sessions) to identify those neurons that carried significant task-related information. Neurons were defined as carrying task-related information if they carried statistically significant stimulus information (defined as in Eq. (1) above), significant choice information (defined as in Eq. (1) above but replacing the stimulus presented in the given trial with the behavioural choice of the animal in the trial), and intersection information. In brief, intersection information uses the mathematical framework of PID [30, 40] to quantify the amount of sensory information encoded in neural activity that is used to inform the behavioural choice [41]. It satisfies a number of information theoretic properties that would be expected of such a measure, including being upper bounded by the stimulus information encoded in neural activity, the choice information encoded in neural activity and by the mutual information between stimulus and choice [40].

The statistical significance of each information measure was computed using a non-parametric permutation test at *p* < 0.1 on the information time-courses. We generated a null hypothesis distribution by randomly shuffling the associations between stimuli and neural responses, or between choices and neural responses, across trials at each time point. For each random permutation, we selected the highest information value across all time windows. We then calculated the *p*-values based on how often the peaks of information in the shuffled dataset exceeded those in the actual dataset. By considering the highest information value as the summary statistic for each trial, the *p*-values obtained as such are already adjusted for multiple comparisons across time. The requirement of all three non-independent tests being satisfied simultaneously was empirically estimated, resulting in a false discovery rate of 1% [34].

We found a subset of 475/2790 neurons that transiently and sequentially carried significant task-relevant information [34]. Using methods described in [26, 34] we next performed GC analysis on this subset of neurons. We selected 20 neurons per session, with peak intersection information exhibiting the shortest latencies. If more than 20 such neurons were present, the 20 with the shortest latencies were selected. We focused our analyses on 12 out of 34 sessions that had at least 20 neurons with significant intersection information. We found that these neurons formed sparse functional networks that transmitted redundant task-relevant information across the trial time (Fig. 1A) [34]. Of these 240 neurons, 144 formed GC connections with at least another neuron (out of the network of 20 neurons that has significant intersection information in the same session) and were termed GC neurons hereafter. The remaining 96 neurons, which did not form GC connections with any other neuron, were termed no-GC neurons hereafter.

We used information-theoretic measures to quantify the stimulus information dynamics of individual neurons. We first considered information in trials in which the mouse made correct perceptual discriminations. The stimulus information time-courses, plotted in (Fig. 1C) after sorting neurons by their peak information timing, showed sequential information coding across the population in both GC and no-GC neurons. At peak, neurons had similar amounts of information in both populations, with the main difference being that GC neurons exhibited the peak information earlier in the trial (during stimulus presentation), whilst no-GC neurons carried information later in the trial (after stimulus presentation) (Fig. 1C). The sequential nature of their activation suggests that information is represented throughout the trial only at the population level, motivating our later information analyses at the neural population level.

To investigate what aspects of neural activity may be a key for correct perceptual judgements, we assessed how information about the auditory stimulus category was encoded in trials in which the animal judged the sound stimulus either correctly or incorrectly. The average stimulus information across all neurons is reported in Fig. 1D. Importantly, we found that the stimulus information was lower in incorrect than in correct trials for both GC and no-GC neurons across the entire trial time, suggesting that the stimulus information is used for the behavioural choice. Importantly, all information quantities computed during correct discriminations were calculated on random subsets of correct trials with the same size as the number of incorrect trials in the same session. Due to this balanced sub-sampling strategy, we were able to make a fair comparison of the amount of information encoded in correct and incorrect trials and control for potential systematic errors due to limited-sampling bias [39].

## Emergent properties of population codes in auditory cortex during correct and incorrect behaviour

We next asked how correct and incorrect behaviour relates to the emergent properties of population codes. This required computing stimulus information from more than 1 neuron.

**Fig. 2:**
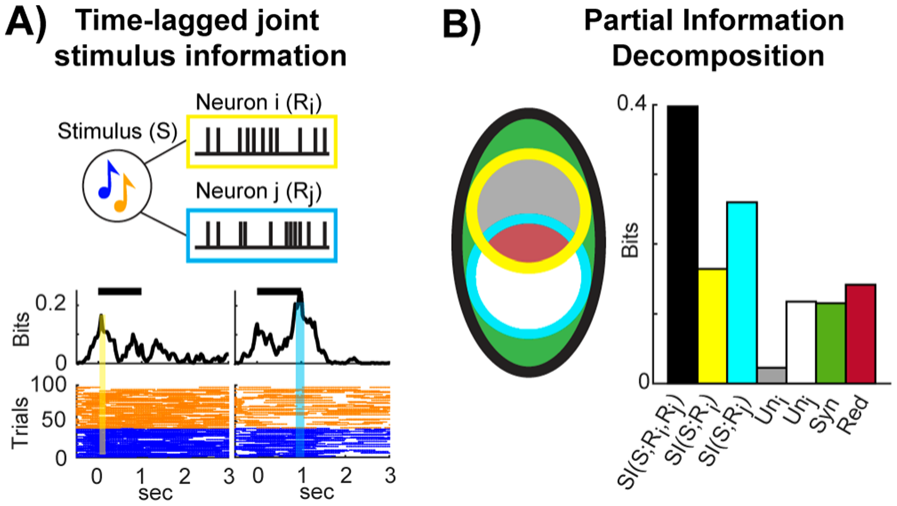
Partial information decomposition of the time-lagged joint stimulus information. **A** Top: Schematic of the computation of the time-lagged joint stimulus information, defined as the mutual information that the neural responses of two neurons at their information time peaks (yellow and cyan vertical bars in the plots on the bottom) jointly carry about the stimulus category. Bottom: The stimulus information time-course (as function of peristimulus time) of two example neurons as a black line, and the deconvolved spiking activity in each trial as orange lines for the low-frequency tones and as a blue line for the high-frequency tones. **B** Left: Sketch as Venn diagram of the decomposition of the joint stimulus information into the non-negative components of synergistic (green area), redundant (red area), and unique (grey and white areas) information. The stimulus information carried by individual neurons (in yellow and cyan) is the sum of the redundant and unique information components. The bar plot on the right shows the amount of the joint stimulus information carried by the example pair of neurons in **A**) (black bar), the stimulus information carried by each neuron individually (yellow and cyan bars), the unique (grey and white bars), synergistic (green bar), and redundant (red bar) information

As in our previous study [34], we estimated the total stimulus information that was jointly carried by pairs of neurons following a time-lagged approach (Fig. 2A). We first identified for each neuron the peak time of task-related information, i.e., the time frame when intersection information time-courses peaked. We then computed the time-lagged stimulus information carried jointly by the activity of each pair of neurons as follows:

2 $$SI\left(S;{R}_{i},{R}_{j}\right)={\sum }_{s\in S, {r}_{i}\in {R}_{i}, {r}_{j}\in {R}_{j}} p\left(s,{r}_{i},{r}_{j}\right)lo{g}_{2}\frac{p\left(s,{r}_{i},{r}_{j}\right)}{p\left(s\right)p\left({r}_{i},{r}_{j}\right)},$$

where $p(s,{r}_{i},{r}_{j})$ denotes the probability of simultaneously observing in the same trial the value *s* of the stimulus category and the joint neural responses ${r}_{i}$ and ${r}_{j}$ of neurons *i* and *j* measured at their respective peaks of task-related information.

First, following our previous work [34], we investigated the nature of redundant and synergistic interactions in pairs of neurons by computing the so-called co-information [42], defined as the difference between the total stimulus information that was jointly carried by both neurons (Eq. (2)) and the sum of stimulus information carried by each neuron individually (Eq. (1)):

3 $$CoInfo\left(S;{R}_{i};{R}_{j}\right)= SI\left(S;{R}_{i},{R}_{j}\right)- \left(SI\left(S;{R}_{i}\right)+ SI\left(S;{R}_{j}\right)\right)$$

A positive value of $CoInfo(S;{R}_{i};{R}_{j})$ implies that the pair of neurons carries more information than the sum of their individual information and can thus be interpreted as predominant synergy. Similarly, a negative value can be interpreted as predominant redundancy.

**Fig. 3:**
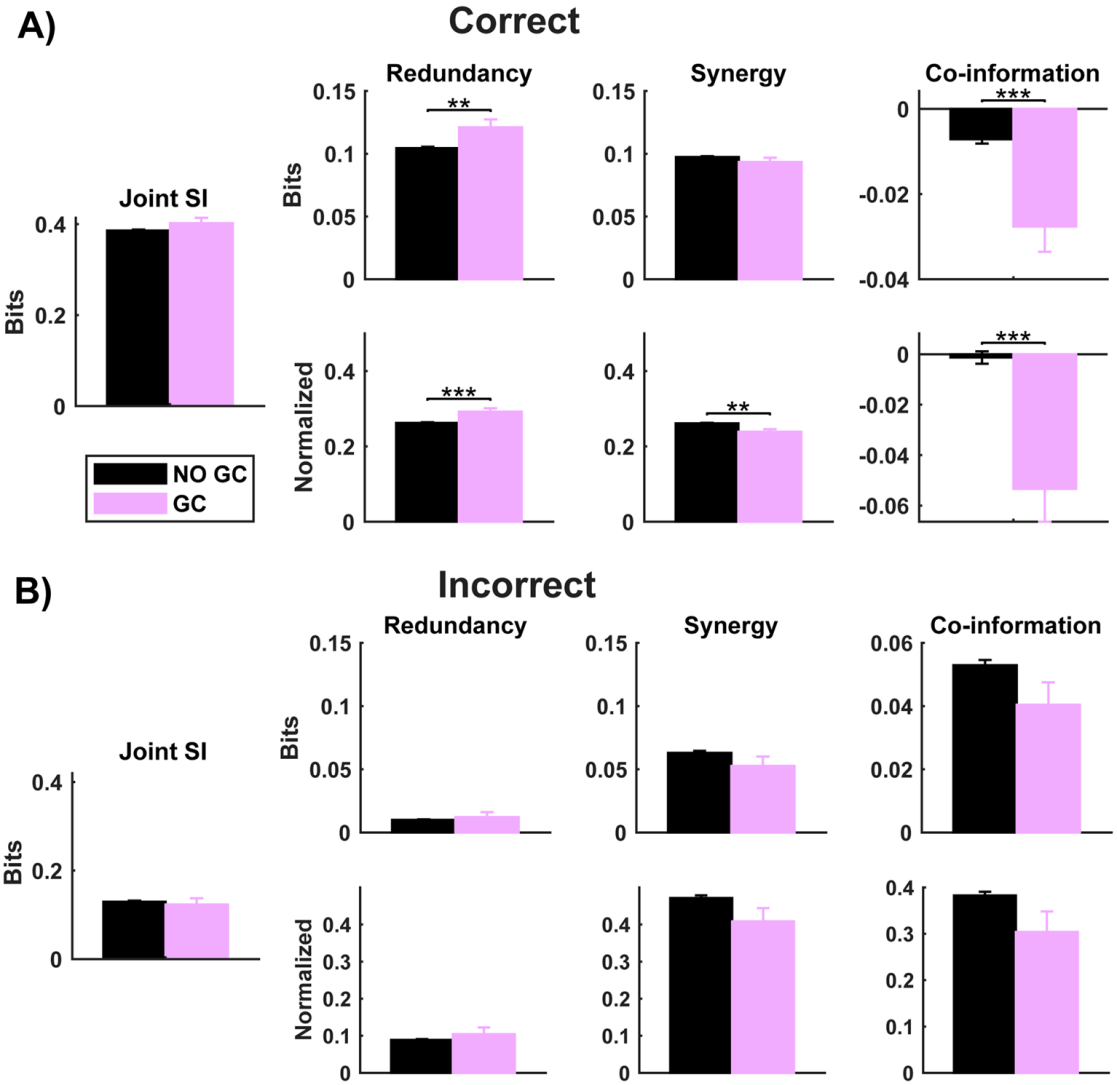
Redundancy and synergy in auditory cortex. **A** Redundancy and synergy computed in correct trials. From left to right: time-lagged joint stimulus information (Joint SI), redundancy, synergy, and co-information for GC-connected (purple) and GC-unconnected pairs of neurons (black). For synergy, redundancy and co-information, the top plots show values in bits and the bottom plots show values normalized by the joint stimulus information. **B** as in panel **A**, but in the case of incorrect trials. Bar plots show mean ± SEM across pairs. Statistics were made with a *t*-test (*p < 0.05, **p < 0.01, ***p < 0.001)

As in our previous study [34], on average across pairs of neurons we found negative co-information (indicating predominance of redundancy) in correct trials and positive co-information (indicating predominance of synergy) in incorrect trials (Fig. 3A).

## Using PID to measure stimulus-related synergy and redundancy in auditory cortex during correct and incorrect behaviour

However, the above results leave the question of how synergy and redundancy separately change between correct and incorrect trials unaddressed. Thus, it is not clear how synergy and redundancy correlate with the accuracy of behavioural decisions.

In fact, it has been shown that co-information conflates two non-negative pieces of information which properly and separately quantify synergy and redundancy [30]. Indeed, there could be cases in which co-information is low, but synergy and redundancy are both high and cancel out due to their opposing signs [43]. In simple terms, redundancy (the area in red in the Venn diagram in Fig. 2B) quantifies the amount of information that both neurons carry independently about the stimulus, whilst synergy (the area in green in the Venn diagram in Fig. 2B) is the amount of information that can be accessed when observing both neuronal responses simultaneously and is not carried individually by any of the two neurons. Thus, the previously reported results could arise in distinct scenarios: redundancy is higher in correct rather than in incorrect trials, synergy is lower in correct than in incorrect trials, or a combination of the two.

To determine the specific contributions of synergy and redundancy to the total joint information, we used the formalism of PID [30]. PID allows breaking down the joint mutual information that two or more source variables carry about a target variable into non-negative and interpretable pieces of information (termed *information atoms*) which quantify how information about the target variable is distributed amongst source variables. In the case of a system with two source variables and one target variable, PID breaks down the joint mutual information encoded by the two sources about the target (See Eq. (2)) into four non-negative information atoms [30, 44]:

4 $$SI\left(S;{R}_{i},{R}_{j}\right)= Red\left(S:{R}_{i},{R}_{j}\right)+Syn\left(S:{R}_{i},{R}_{j}\right)+ U{n}_{i}\left(S:{R}_{i}\backslash {R}_{j}\right)+U{n}_{j}\left(S:{R}_{j}\backslash {R}_{i}\right),$$

where $Red(S:{R}_{i},{R}_{j})$ is the redundant information (red area in the Venn diagram in Fig. 2B) which is present in both neuron ${R}_{i}$ and neuron ${R}_{j}$, $Syn(S:{R}_{i},{R}_{j})$ is the synergistic information (green area in Fig. 2B) carried only by the joint response of the two neurons, whilst $U{n}_{i}\left(S:{R}_{i}\backslash {R}_{j}\right)$ and $U{n}_{j}(S:{R}_{j}\backslash {R}_{i})$ stand for the two unique information components (grey and white areas in Fig. 2B, respectively) carried by one source variable but not by the other. Importantly, the four information atoms appearing in the right-hand side of Eq. (4) are not independent, so that determining the value of one atom is sufficient to compute all the others, as the other three can be computed as linear combinations of Shannon information-theoretic quantities and the determined atom [44].

An important insight arising from the PID is that $CoInfo(S;{R}_{i};{R}_{j})$ given in Eq. (3) is the difference between the two distinct information atoms that express synergy and redundancy, respectively:

5 $$CoInfo\left(S;{R}_{i};{R}_{j}\right)= Syn\left(S:{R}_{i},{R}_{j}\right)- Red\left(S:{R}_{i},{R}_{j}\right).$$

To compute $Red(S:{R}_{i},{R}_{j})$ and $Syn(S:{R}_{i},{R}_{j})$ we used the definition provided by [44]. Given a trivariate probability distribution $P(S,{R}_{i},{R}_{j})$, Bertschinger et al. defined the unique information atom as follows [44]:

6 $$U{n}_{i}\left(S:{R}_{i},{R}_{j}\right)=\underset{Q\in {\Delta }_{P}}{\mathrm{min}}{I}_{Q}\left(S:{R}_{i}\backslash {R}_{j}\right),$$

which defines a constrained convex optimization problem in the space ${\Delta }_{P}$ of trivariate probability distributions $Q(S,{R}_{i},{R}_{j})$ with fixed marginals $Q\left(S,{R}_{i}\right)=P(S,{R}_{i})$ and $Q\left(S,{R}_{j}\right)=P(S,{R}_{j})$.

To numerically solve this optimization problem, we used the *BROJA_2PID* python package [45]. In this way, we computed the synergistic and the redundant information that pairs of neurons carried about the stimulus.

Following [34], we labelled the neuronal pairs as GC-connected if they shared at least one GC link and as GC-unconnected otherwise. We performed the stimulus-related PID analysis in the two separate groups of GC-connected and GC-unconnected pairs of neurons in correct and incorrect trials.

We first performed the PID analysis in correct trials for both the GC-connected and GC-unconnected pairs of neurons (Fig. 3A). To obtain a fair comparison between results in correct and incorrect trials, we performed these analyses over a randomly selected subsample of correct trials with the same sample size as the incorrect trials (results are presented as average over 100 random subsamples). We found that the joint stimulus information had comparable values (0.386 ± 0.002 bits vs 0.402 ± 0.012 bits for GC-unconnected vs GC-connected pairs respectively; hereafter, all results in this section are reported as mean ± SEM over all pairs of neurons) in both populations. However, GC-connected pairs had higher levels of redundancy (0.121 ± 0.006 bits) compared to the GC-unconnected ones (0.105 ± 0.001 bits), whilst they had similar amounts of synergy for GC-unconnected (0.097 ± 0.001 bits) and GC-connected pairs (0.093 ± 0.001 bits) respectively (Fig. 3A). Confirming the previously reported results [34], the difference between synergy and redundancy, i.e., the co-information (Eq. (5)), showed a prevalence of redundant information in both populations, but the GC-connected pairs were more redundant (-0.027 ± 0.006 bits) than GC-unconnected pairs (-0.007 ± 0.001 bits).

We next quantified the fraction of redundancy and synergy by normalizing each term with respect to the total joint mutual information. This is useful to discount any possible effect of differences in information levels between correct and error trials. We found that GC-connected pairs had proportionally more redundancy and less synergy (Red = 0.292 ± 0.010, Syn = 0.239 ± 0.007), compared to GC-unconnected ones (Red = 0.262 ± 0.001, Syn = 0.261 ± 0.001) (Fig. 3A). Moreover, GC-connected pairs had much more predominant redundancy (− 0.053 ± 0.013) than GC-unconnected pairs (− 0.001 ± 0.002).

In sum, our results suggest that GC-connected pairs of neurons have more redundant than synergistic functional connections.

Next, we investigated whether higher amounts of redundancy and lower amounts of synergy could be beneficial for task performance and behavioural accuracy. We computed the PID in incorrect trials (Fig. 3B). The joint stimulus information in incorrect trials (0.130 ± 0.002 bits, 0.123 ± 0.014 bits for GC-unconnected and GC-connected pairs respectively) was only ~ 30% of what it was in correct trials. Redundancy in incorrect trials had a value of 0.010 ± 0.001 bits, 0.012 ± 0.004 bits for GC-unconnected and GC-connected pairs respectively, which is proportionally 10 times smaller than that of correct trials. Synergy dropped to 0.063 ± 0.002 bits and 0.053 ± 0.007 bits for GC-unconnected and GC-connected pairs respectively, proportionally only half of that in correct trials. Co-information showed positive values, i.e., more synergy than redundancy, in both GC-unconnected (0.053 ± 0.002 bits) and GC-connected pairs (0.040 ± 0.007 bits). Normalized redundancy constituted approximately 10% of the total information, whereas normalized synergy amounted to ~ 45% (Fig. 3B). We did not find significant differences in the normalized co-information between GC-unconnected and GC-connected pairs on incorrect trials (0.382 ± 0.008 vs 0.304 ± 0.044). Our results suggest that only the redundant FC associated with GC links is beneficial to correct sensory discrimination.

## Predicting correct vs incorrect perceptual discriminations based on redundancy and synergy of functionally connected neurons

Given that GC-connected pairs of neurons exhibit higher values of normalized redundancy and lower values of normalized synergy during correct decisions compared to incorrect ones, we sought to determine whether redundancy or synergy is more predictive of the correctness of perceptual discriminations. We focused this analysis on normalized redundancy and synergy values to control for potential confounding effect of differences in joint information values between correct and incorrect trials.

**Fig. 4:**
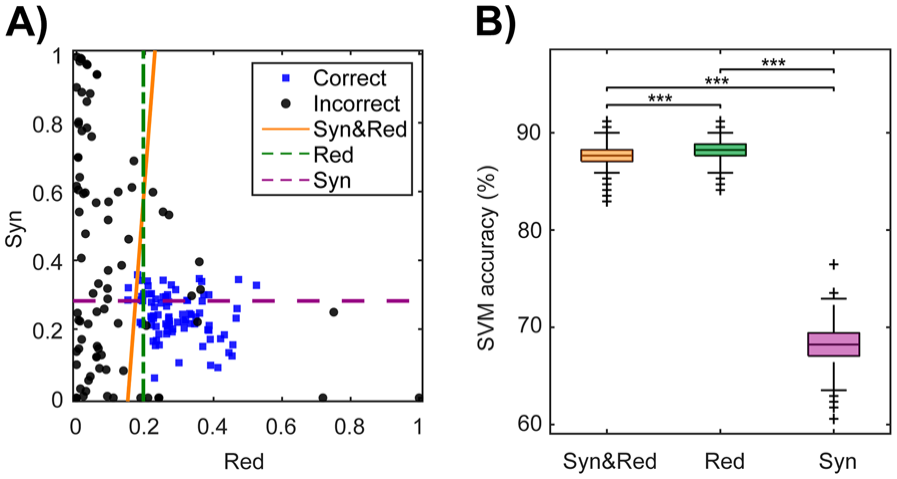
Decoding behavioural decisions through normalized synergy and redundancy in GC-connected pairs.** A** We used normalized synergy and redundancy to classify behavioural decisions as correct (blue squares) or incorrect (black dots) perceptual discriminations. The solid orange line is the decision boundary of an SVM trained using both synergy and redundancy values as predictive features. Green and purple dashed lines are the decision boundaries of SVM trained using only redundancy or synergy, respectively, as a predictive feature. Correct decisions are characterized by clustered values in both synergy and redundancy, whilst incorrect decisions show lower redundancy values and a uniform distribution of synergy. **B** SVM cross-validated classification accuracy from 10,000 bootstrap samples. Colours of the distributions match the ones of decision boundaries in **A**. Statistical comparisons across different models were made using the Wilcoxon rank-sum test (***p < 0.001)

To visualize this dependency in an intuitive way, in Fig. 4A we present a scatterplot (on the n = 85 pairs of GC-connected neurons that were used in previous analyses) of how normalized synergy and redundancy values are distributed across GC-connected pairs for correct and incorrect decisions. Visual inspection of this plot suggests that normalized redundancy values have a strong discrimination power, with high values of normalized redundancy predicting correct choices and low values of normalized redundancy predicting incorrect choices. To support this intuition with quantitative analyses, we used a soft-margin Support Vector Machine (SVM) with a linear kernel to discriminate between correct and incorrect behavioural decisions from the normalized synergy and redundancy values of GC-connected pairs. Specifically, we used the MATLAB function *fitcsvm* with default arguments, in particular with regularization parameter C = 1 and optimization with the Sequential Minimal Optimization algorithm, and we used a leave-one-out cross-validation procedure on the n = 85 pairs of GC-connected neurons. We first classified correct vs incorrect behaviour when the SVM used both normalized redundancy and synergy and found that, when using both features, it yielded a high classification accuracy of 87.60 ± 1.20% (hereafter, in this section values are reported as mean ± SD across 10,000 bootstrap samples) for correct vs incorrect decisions (Fig. 4B). The SVM weight corresponding to the normalized redundancy had a higher absolute magnitude than the one corresponding to the normalized synergy (magnitude of the redundancy SVM weight: 5.79 ± 0.05; magnitude of the synergy SVM weight: − 0.65 ± 0.04). These results indicate that proportion of redundancy has a major predictive power for the correctness of behavioural decisions.

To further assess the roles of normalized synergy and redundancy values in predicting correct decisions, we used the SVM to classify error vs correct behaviour using each feature separately. The classifier using exclusively on normalized redundancy discriminated correct vs incorrect decisions with a high accuracy (88.28 ± 0.97%), even slightly higher than the classification accuracy achieved considering both normalized synergy and redundancy (Fig. 4B). In contrast, the SVM classifier relying solely on normalized synergy achieved a lower accuracy (67.98 ± 1.77%) than both the classifiers relying solely on synergy or on both synergy and redundancy (Fig. 4B). This shows that once redundancy is known, synergy does not add predictive power about the correctness of perceptual discriminations.

## Discussion

In this study, we teased apart the relationship between FC and stimulus-related synergy and redundancy with single-neuron resolution in the mouse auditory cortex during a perceptual discrimination task. We deliberately considered one specific, widely used type of directed FC measure, Granger Causality. GC is a directed measure, and as such it can disambiguate between stronger information transfer in one direction that the opposite direction. It is a data-robust linear version of the corresponding information theoretic quantity, Transfer Entropy (TE) [46]. Whilst TE has the advantage of possibly capturing non-linear information transfer and it can be also framed in the context of PID [28, 47], the data-robustness of GC allows its easier application in multivariate settings to condition away the effects of other neurons [26]. Importantly for the present study, unlike other measures such as the Pearson correlation between the activity of two neurons, GC can in principle be related to both redundancy and synergy.

Our findings revealed that Granger FC between A1 L2/3 neurons was accompanied by proportionally higher levels of redundancy and lower levels of synergy compared to pairs of neurons that were not linked with a Granger FC. These results suggest that FC creates prevalent redundancy of sensory information across neurons.

Previous work has established that the sensory information encoded by neuronal populations greatly decreases when animals make incorrect perceptual decisions, compared to when animals make correct decisions [17, 48–52]. However, less is still known about how the interactions between neurons in a population code, and the patterns of synergy and redundancy that may be created by these interactions, promote correct decisions [4]. Here, we made progress in this direction by studying not only how information levels change between correct and incorrect trials but also studying patterns of synergy and redundancy. Our results suggest that both synergy and redundancy coexist across the population, both when mice make correct or incorrect perceptual discriminations. However, we found that the levels of redundancy were much higher (both in absolute terms and in proportion to the total information available in neuron pairs) in both populations when mice made correct behavioural choices compared to incorrect ones, whereas synergy values were higher in absolute terms but lower in relative terms during correct compared to incorrect behavioural choices. Moreover, the proportion of redundancy more reliably predicted perceptual discriminations, whilst the proportion of synergy had a much lower predictive power per se, and did not add predictive power once the proportion of redundancy was known.

Overall, the above results suggest that redundancy is highly beneficial for correct sensory judgements. The advantages of redundancy for perceptual discrimination could arise from multiple contributions. One well-documented advantage regards the integration of information across sites [53]. Another one could result in advantages in terms of information transmission and readout. Indeed, whilst redundancy limits the amount of encoded information [54], it has benefits in terms of improving the propagation of information between pre- and post-synaptic neurons [4, 17]. Together with those reported in previous studies [4, 17, 34], our results suggest that the optimal trade-off between the advantages and disadvantages of redundancy results in an overall advantage of having some degree of redundancy to secure reliable downstream information transmission.

Our findings confirm previous reports of significant synergy between the activity of neurons or networks [14, 15, 33]. Our finding that synergy is higher in absolute terms during correct behaviour suggest that synergy may promote correct decisions by elevating the information levels in correct trials. However, our observations of a decreased proportion of synergy during correct perceptual discrimination suggests that the potential advantage of synergy in terms of higher levels of sensory information encoding may not entirely translate into an advantage for sensory discrimination. One possibility is that the interactions leading to synergistic information may be more difficult to be read out by downstream computations, as they would require more sophisticated decoders that may be beyond the capabilities of some downstream neural circuits. However, given that presence of synergy has been well-documented, another possibility, to be explored in future studies, is that synergy may not be needed for the simple perceptual tasks we consider and for neurons in sensory areas, but that it could become more important for more complex behaviours or for neurons in higher level areas [14].

Together, these results establish the major importance of redundancy amongst neurons in sensory cortices for correct sensory discriminations which may be due to the beneficial effects that redundancy has on downstream information transmission. At the same time, our results suggest also a smaller yet useful contribution of synergy to correct perceptual discriminations, by enhancing information levels during correct behaviour.

Another important question regards how synergistic and redundant FC relate to structural connectivity [14]. Robust and meaningful relationships have been established between redundant FC measured during the resting state and structural connectivity at the level of whole-brain measures that lack cellular resolution [14, 21]. However, it remains to be understood how this anatomical substrate is complemented by stimulus-dependent changes in neural dynamics. The same structural connectivity can give rise to different patterns of functional connectivity depending for example on the state of each node. For example, depending on the degree of excitability of a given node, the functional interactions between areas can be larger or smaller even if the anatomical connections between them do not change. As a result, the relationship between functional and structural connectivity is complex [55] and changes in state of individual nodes or on the stimulus information present in the inputs to some of the considered nodes can modulate both redundancy and synergy between anatomically connected nodes. Detailed studies of realistic neural network models, as well as careful experiments that manipulate the activity of individual nodes [56], will be a key to progress in addressing these questions.

From a theoretical perspective, previous studies that investigated synergy and redundancy between neurons or networks employed a measure of co-information which conflates synergy with redundancy, measuring only their net effect [32, 34]. Our work advances the state-of-the-art by providing a more refined measure that delineates redundancy from synergy and enables separate quantification of their relationship with both FC and the accuracy of behaviour. With respect to other studies considering redundancy and synergy, but not relating it to information content about variables of cognitive interest [14], we made progress by measuring redundancy and synergy of information about variables, such as sensory stimuli, which have a well-defined meaning and role in terms of perceptual functions. We hope that our work will contribute to creating a neuroinformatics framework that can help researchers to study the patterns of synergy and redundancy about external stimuli and pinpoint their contribution to behaviour and functions. This progress will need to include mathematical advances in the understanding of the differences and complementarity between different possible PID formalisms. For example, the formalism we used here [44] breaks down information into non-negative parts, as in the PID original formulation [30, 47]. However other work is exploring the advantages of alternative ways to decompose information, including decompositions into terms that do not need to be non-negative [57–60]. It would also be important to understand the relationship between the PID-based formalisms and previously derived information-theoretic formalisms that quantify how the information in a population of neurons depends on the correlations of the activity of different neurons [32, 61–63]. These previous studies established important rules for how correlations between neurons can enhance or decrease information and change co-information values (for example, correlations can increase information and thus create synergy, when their strength is modulated by the stimulus). Connecting these formalisms will aid the understanding of how synergy and redundancy may arise in terms of basic properties of neural activity or of circuit mechanisms.

In conclusion, our study provides a framework to measure the behavioural relevance of synergy and redundancy even with cellular resolution. The results obtained analysing the activity of auditory cortex with this framework suggest that correct behaviour is associated with a predominant presence of redundant information in functionally connected neural networks. Further research is needed to better understand the contributions of synergy and redundancy in different contexts.

## Data Availability

The code used to compute information measures is taken from [40] and can be downloaded at https://doi.org/10.5281/zenodo.850362. The experimental data were shared in a previous publication [34] and can be downloaded from the Digital Repository at the University of Maryland at https://drum.lib.umd.edu/items/30d43732-7149-4726-a860-0ae3d210b2ae. Any additional information required to reanalyse the data reported in this paper is available from the corresponding authors upon reasonable request.

## Abbreviations

FC: Functional connectivity
fMRI: Functional Magnetic Resonance Imaging
EEG: Electroencephalogram
GC: Granger causality
PID: Partial Information Decomposition
A1: Primary auditory cortex
L2/3: Layer 2/3
SEM: Standard Error of the Mean
SI: Stimulus information
CoInfo: Co-information
Red: Redundancy
Syn: Synergy
Un: Unique information
SVM: Support Vector Machine
SD: Standard deviation
TE: Transfer entropy
